# Global Spectral Analysis of Polaritonic Coupling of Multiple Organic Dyes to a Tunable Fabry‐Pérot Resonator Operating with Mirror Separations up to 10 μm

**DOI:** 10.1002/chem.202500344

**Published:** 2025-03-05

**Authors:** Christoph Kertzscher, Michael Mauch, Jakob Keck, Alfred J. Meixner

**Affiliations:** ^1^ Institute of Physical and Theoretical Chemistry University of Tübingen Auf der Morgenstelle 18 72076 Tübingen Germany

**Keywords:** Donor-acceptor systems, Fabry-Pérot resonator, J-aggregate, Photophysics, Polariton

## Abstract

We study strong optical coupling and the formation of hybrid polaritons by a donor‐acceptor pair of J‐aggregated dyes separated by up to 11 μm in a tunable Fabry‐Pérot resonator. Fitting a phenomenological Hamiltonian to the white light transmission spectra of different cavity‐J‐aggregate‐configurations, for which several hundred spectra were recorded sequentially as function of cavity length, allows us to determine coupling energies as a function of the effective cavity distances. The theoretical analysis shows that certain hybrid polaritons contain contributions from both dyes, making this system a promising platform for polariton‐mediated energy transfer at very large distances.

## Graphical abstract

We demonstrate simultaneous strong coupling of two different J‐aggregated dyes embedded in a tunable Fabry‐Pérot resonator at mirror distances exceeding 10 μm. The observed polariton modes can be modeled by a phenomenological Hamiltonian. Even at very large distances, there are hybrid polaritons present, which contain contributions from both dye molecules, making this system a promising platform for studying polariton‐mediated energy transfer.

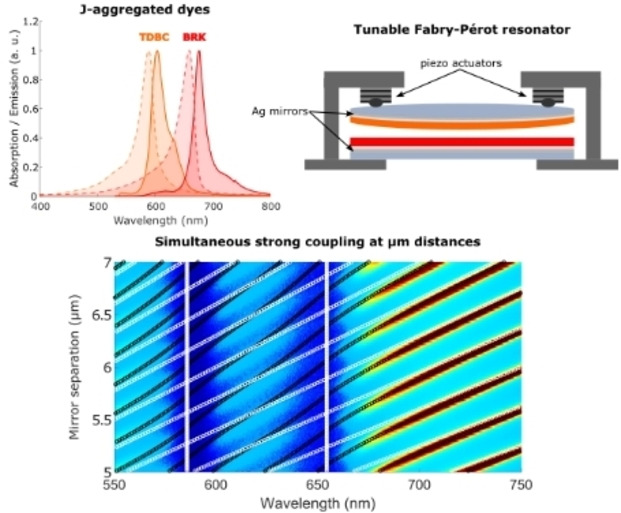

## Introduction

After the first demonstration of strong coupling in organic molecules embedded in an optical resonator, which was achieved by Lidzey *et al*. in 1998,^[1]^ hybrid light‐matter states called (cavity) polaritons have created considerable attention since they possess new characteristics that are not found in either the original exciton or the optical mode. As a result, material properties such as energy transfer,[^2^, ^3^, ^4^, ^5^, ^6^, ^7^, ^8^, ^9^] transport processes,[^10^, ^11^, ^12^, ^13^, ^14^, ^15^] or chemical reactivity,[^16^, ^17^, ^18^, ^19^, ^20^, ^21^] may undergo significant changes.[^22^, ^23^, ^24^, ^25^, ^26^, ^27^] These states can emerge if an optically active material, characterized by its excitonic transitions, is enclosed in an optical Fabry‐Pérot (FP) microcavity. If the energy exchange between the excitons and the optical modes of the microcavity is faster than competing dissipation processes, *i. e*. is reversible, the system is said to be strongly coupled and new eigenstates are formed.

In previous works, cavities were typically constructed by successive thin film preparation steps (substrate – Ag mirror – active layer – spacer layer – active layer – Ag mirror), resulting in rigid FP resonators with fixed distances between the two mirrors. For such systems, the energy of the cavity modes can be tuned by varying the incidence angle of the radiation and performing angle‐resolved measurements, which exploit the parabolic dispersion relation $E\left(k\right)$ of the cavity modes.^[28]^ We, on the other hand, use an open FP resonator with tunable mirror separation.[^29^, ^30^, ^31^, ^32^, ^33^, ^34^, ^35^, ^36^, ^37^, ^38^] This enables us to increase the mirror distance continuously by several μm during a single measurement and to investigate the system's behaviour as a function of cavity length at mirror separations exceeding 10 μm, which is much larger than previously reported.

If several different excitons are coupled to the same microcavity, the resulting hybridisation has been shown to enable “Polariton‐mediated energy transfer” (PMET)[^2^, ^3^, ^4^, ^5^, ^6^] at much larger distances (up to 2 μm^[6]^) than conceivable *via* dipole‐dipole interactions (Förster^[39]^) or electronic exchange (Dexter^[40]^), which are inherently short‐ranged. For the mechanistic explanation of PMET, previous authors stressed the importance of “hybrid polaritons”[^2^, ^3^, ^4^, ^5^, ^6^] which contain significant contributions from both excitonic constituents.

**Figure 1:**
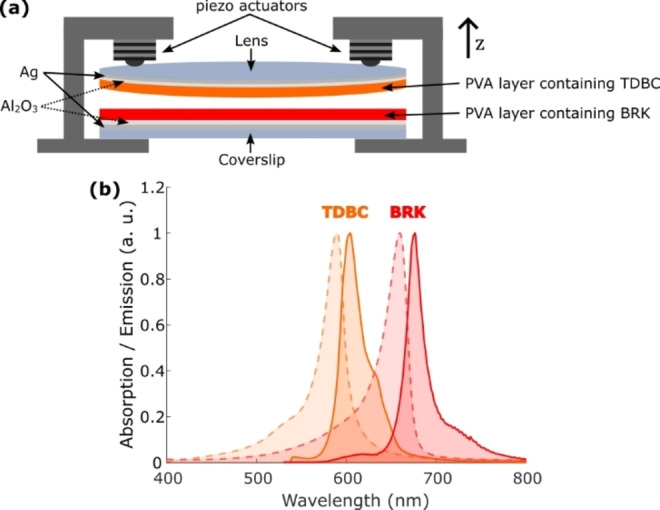
(a) Sketch of the tunable FP resonator. (b) Free space absorption (dashed lines) and emission spectra (solid lines) of the two J‐aggregates TDBC (orange) and BRK (red).

In this work, we study the formation of strong coupling and hybrid polaritons by a donor‐acceptor pair of J‐aggregated dyes separated by up to 11 μm in a tunable FP resonator. Thin films containing the well‐known J‐aggregates TDBC as energy donor and BRK as energy acceptor were deposited on top of the adjacent mirrors (film thickness 500–600 nm, see SI), as sketched in Figure 1(a). J‐aggregates are organic molecules with very favourable optical properties due to supramolecular self‐organization,^[41]^ such as intense and narrow J‐bands and a small Stokes shift as shown in Figure 1(b) by the TDBC and BRK absorption and emission spectra, making them ideal for achieving strong coupling at room temperature.^[42]^ We present experimental white light transmission spectra as a function of the mirror separation up to 12 μm for different cavity‐J‐aggregate configurations, which were successfully fitted by a phenomenological Hamiltonian, which allowed us to determine the coupling energies as a function of the effective optical cavity length.

## Results and Discussion

### Transmission Measurements

We prepared three different cavity‐J‐aggregate‐configurations: the donor TDBC on the top mirror (2M‐D), the acceptor BRK on the bottom mirror (2M‐A), and a donor‐acceptor configuration with TDBC on the top mirror and at the same time BRK on the bottom mirror (2M‐DA‐I). To ensure comparability we used the same coated mirrors for all three configurations. After setting up a certain configuration, we brought the top mirror into contact with the bottom mirror. Then, we used the piezo actuators to stepwise move the upper mirror in the z ‐direction by gradually decreasing the applied voltage, which increased the cavity distance, until the minimum voltage was reached giving a mirror separation of about 5 μm. Following each voltage step, we recorded a transmission spectrum. For the configuration involving both J‐aggregates (2M‐DA‐I), we moved the upper mirror to the approximate endpoint of the first measurement using μm‐screws to increase the starting cavity distance. We then performed a second scan, moving the piezo actuators over the whole voltage span a second time at larger distances of approximately 5–10 μm (2M‐DA‐II).

**Figure 2:**
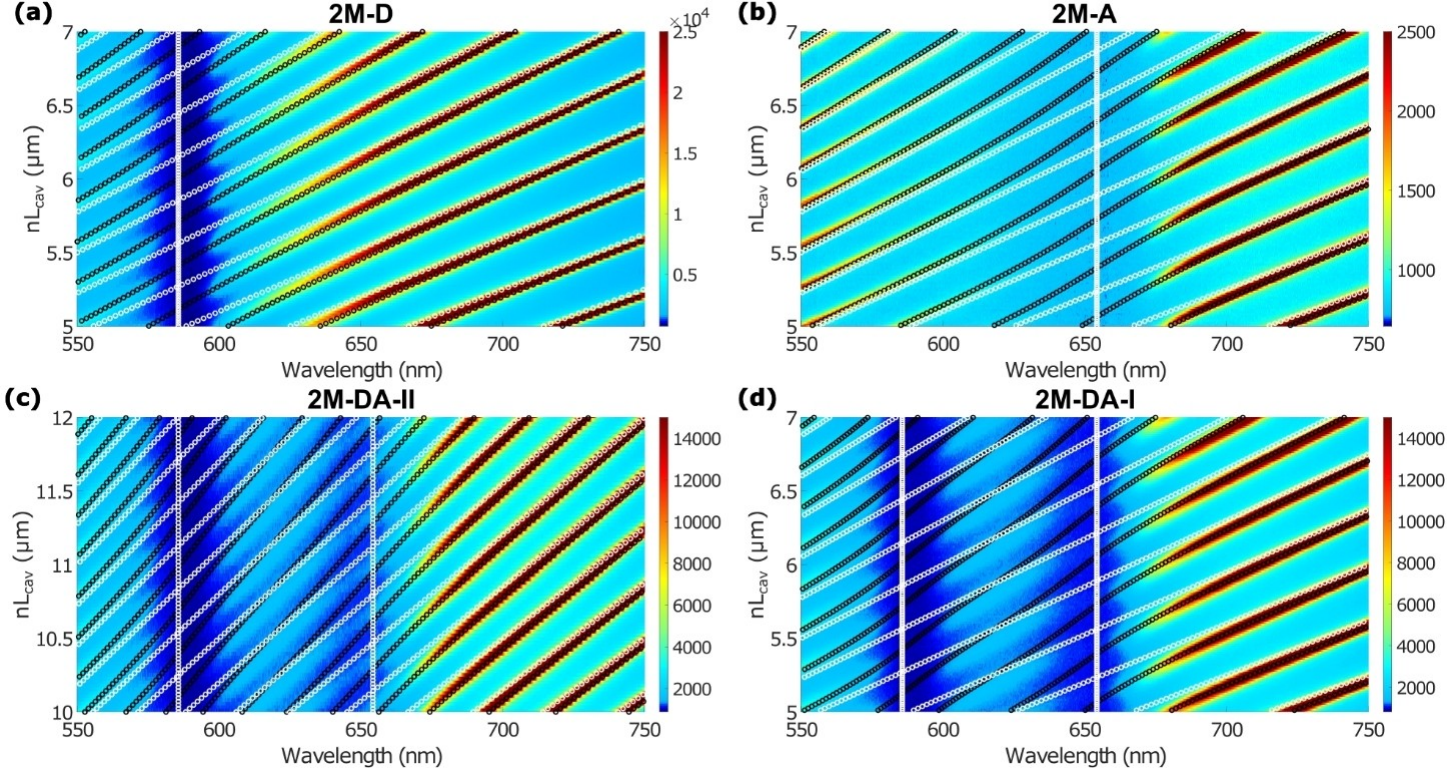
Transmission spectra plotted as false‐color maps for different cavity‐J‐aggregate‐configurations: Only TDBC in (a), only BRK in (b), both TDBC and BRK in (c) and (d). Black circles show the fits to $H_{coupled}$ . For comparison, white circles show the corresponding uncoupled excitons and cavity modes. The spectra are plotted against the cavity distances from the fits. Here, we only show cutouts of the obtained maps for better visibility. Maps showing the entire cavity tuning range are provided in the SI.

In Figure 2, we present the series of transmission spectra plotted as false‐color maps. Here we only show smaller sections for better visibility. The complete false‐color maps are provided in the SI. For strongly coupled J‐aggregate thin films, usually only a small fraction of the aggregates couples to the light field while most remain uncoupled.[^43^, ^44^, ^45^, ^46^] Hence, the transmission around the present excitonic transitions (TDBC and/or BRK) is very low, which is due to absorption by uncoupled molecules. The absolute intensities of the transmission peaks change with wavelength. This is mainly due to the spectrum of the used white light source, which we did not correct for.

From the progression of the transmission maxima, it is already evident that we are operating in the strong coupling regime since the transmission maxima do not follow straight lines as would be expected for an uncoupled cavity (see Equation (1) and Figure S2) but show clear bending near the excitonic transitions.

### Theoretical Model

The spectral evolution of the transmission spectra as a function of the cavity length can be interpreted in terms of a theoretical model based on strong exciton‐photon coupling. For this purpose, we have developed a procedure to fit the experimentally obtained transmission maxima by the phenomenological Hamiltonian outlined below. The results from the fits are plotted as black circles in Figure 2. The phenomenological Hamiltonian contains the exciton energies of the J‐aggregates $E_{TDBC}$ and $E_{BRK}$ as well as the photon energies of the cavity modes as diagonal elements, determined *via*

(1) $$E_{cav,m}=m\frac{\pi \hbar c}{nL_{cav}}\;with\;m=1,2,3,\;\cdots ,$$

where m is the mode number, ℏ is the reduced Planck's constant, c is the vacuum speed of light, and $nL_{cav}$ is the optical path length. The exciton‐photon coupling energies $\hbar g_{TDBC}$ and $\hbar g_{BRK}$ which correspond to one half of the Rabi splitting energy are introduced as off‐diagonal elements. For the exciton energies we choose $E_{TDBC}$ =2.117 eV (corresponding to 585.59 nm) and $E_{BRK}$ =1.895 eV (corresponding to 654.29 nm) which are the absorption maxima of TDBC and BRK, determined from Lorentzian fits to the free space absorption spectra (see Figure 1(b)). We chose these values since it was demonstrated that for systems incorporating porphyrins for which absorption and emission are clearly distinguishable (large Stokes shift), strong coupling was achieved with the absorption band.[^1^, ^47^] The optical path length and the relevant coupling energies are treated as parameters, which are fitted to the experimental data.

The different energies are arranged in a Hamiltonian matrix (labelled “$H_{coupled}$ ”, left most matrix in Equation (2)). The energies of the resulting polariton modes are then simply the eigenvalues of this matrix.

The hybrid light‐matter states are characterized by coefficients, which indicate the fraction or relative contribution of the cavity modes and the excitons (sometimes called “Hopfield coefficients”). To obtain these coefficients, we explicitly write down the eigenvalue equation for $H_{coupled}$ :
(2)

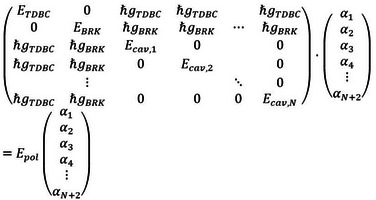




Here, the vector with entries $\alpha_{i}$ is the eigenvector and $E_{pol}$ is the eigenvalue, which corresponds to the energy of a certain polariton mode. N is the number of considered cavity modes. By solving this equation, one obtains N+2 eigenvalues $E_{pol}$ with associated eigenvectors $\left(\alpha_{i}\right)$ .

For the uncoupled system, $\hbar g_{TDBC}$ =$\hbar g_{BRK}$ =0 and $H_{coupled}$ becomes a diagonal matrix. Consequently, the eigenvalues are just the diagonal elements $E_{TDBC}$ , $E_{BRK}$ , $E_{cav,1}$ , …, $E_{cav,N}$ and the eigenvectors are just the trivial base vectors. Therefore, each $\alpha_{i}$ directly relates to an eigenvector in the uncoupled basis and can be associated with a certain exciton transition or cavity mode: $\alpha_{1}↔E_{TDBC}$ , $\alpha_{2}↔E_{BRK}$ , $\alpha_{3}↔E_{cav,1}$ , $\alpha_{4}↔E_{cav,2}$ , …

The eigenvectors are normalized such that the absolute values squared ${\left|\alpha_{i}\right|}^{2}$ are a good measure for the contribution of the different constituents of the polariton ($0\leq {\left|\alpha_{i}\right|}^{2}\leq 1$ and $\sum_{i}{\left|\alpha_{i}\right|}^{2}=1$ ).

In our system, we have two exciton parts (the two J‐aggregates TDBC and BRK), which are characterized by the first two entries of the eigenvectors:

(3) $$\begin{array}{c} {\left|\alpha^{TDBC}\right|}^{2}={\left|\alpha_{1}\right|}^{2}\\ {\left|\alpha^{BRK}\right|}^{2}={\left|\alpha_{2}\right|}^{2} \end{array}$$

For the light part, we look at the remaining vector elements and sum them up for simplicity:

(4) $${\left|\alpha^{light}\right|}^{2}=\sum_{i=3}^{N+2}{\left|\alpha_{i}\right|}^{2}$$

### Fitting Procedure

To fit the transmission spectra by the phenomenological Hamiltonian, we have developed a fitting procedure in MATLAB (details are provided in the SI) which allows us to fit the entire series of transmission spectra as a function of increasing mirror separation with the effective optical path lengths of the cavity and the coupling energies as fit parameters. The resulting polariton modes are shown as black circles in Figure 2. The cavity lengths increase slightly faster than linear over the course of the entire series of spectra for all investigated cavity configurations (see Figure S4) while the coupling energies ℏg decrease with increasing cavity length (see Figure S5) as expected for a Fabry‐Pérot resonator with cavity volume V :[^48^, ^49^]

(5) $$\hbar g=\sqrt{\frac{\hbar \omega }{2\epsilon_{0}V}}\mu .$$

Here, μ is the transition dipole moment of the embedded material, ω is the angular frequency of the cavity mode, and $\epsilon_{0}$ is the vacuum permittivity. Assuming $V\propto nL_{cav}$ , we expect a decrease of the coupling energy as $\hbar g=A\cdot {\left(nL_{cav}\right)}^{-1/2}$ , where A is a material‐dependent constant which we treated as a fit parameter. By adjusting A , we can describe the obtained coupling energies with very good agreement for distances >3.5 μm. Small systematic deviations we observe in the obtained curves (Figure S5) are discussed in the SI. The values for the coupling strength and for the optical path lengths obtained from the fits are listed in Table 1.

**Table 1:** Results for the fit parameters (“coupling strength” A and optical path length $nL_{cav}$ ) from the best fits of $H_{coupled}$ to the transmission spectra. The coupling energy ℏg is related to A *via* $\hbar g=A•{\left(nL_{cav}\right)}^{-1/2}$ . In the last column, we also indicate the distance between the two J‐aggregate‐containing layers (by assuming that the layers touched at the start of the measurement).

	Coupling strength A (eV (nm)^−1/2^) for $\hbar g_{TDBC}$	Coupling strength A (eV (nm)^−1/2^) for $\hbar g_{BRK}$	Range of $nL_{cav}$ (μm) during measurement	Distance between J‐aggregates (μm)
2M‐D	5.5	–	2.456 … 7.850	–
2M‐A	–	4.0	1.138 … 7.040	–
2M‐DA‐I	3.5	3.5	1.433 … 7.210	0 … 5.8
2M‐DA‐II	3.5	3.5	7.260 … 12.800	5.8 … 11.4

In Table 1, we also calculated the distances between the J‐aggregate containing layers for 2M‐DA‐I and 2M‐DA‐II by assuming that the mirrors touched at the start of 2M‐DA‐I (for further discussion on this point, see the SI).

There is some debate in the literature regarding the appropriate Hamiltonian for strongly coupled multimode cavities. Some works[^6^, ^50^] recommend the “decoupled” Hamiltonian $H_{decoupled}$ where each cavity mode is coupled to the excitons independently (see SI). Alternatively, a transition between $H_{decoupled}$ and the “coupled” Hamiltonian $H_{coupled}$ (left most matrix in Equation (2)) where the excitons couple with each cavity mode simultaneously is proposed depending on the oscillator strength of the active layer.[^51^, ^52^]

Therefore, we have fitted both Hamiltonians to the transmission data using the procedure outlined above and found significantly better agreement for $H_{coupled}$ (more details are provided in the SI). This result is consistent with the findings of Georgiou *et al*. who have investigated strong coupling of spin coated thin films containing TDBC in an optical microcavity.^[51]^ They observed for low‐concentration TDBC solutions (1 and 2 wt %) that $H_{coupled}$ matched the experimental data better, while for high‐concentration TDBC solutions (4 and 6 wt %) $H_{decoupled}$ matched the experimental data better.

### Hybrid Polaritons

Using the fit results along with the mathematics outlined above (Equations (3) and (4)), we can calculate the light‐, TDBC‐ and BRK‐contributions for the obtained polariton modes. Therefore, we can investigate if there are hybrid polaritons present in the investigated system. We found for most cases that there is at least one hybrid polariton with energy in between the TDBC and BRK exciton energies with at least two percent excitonic contribution from TDBC and BRK. The only exceptions were configurations for which no uncoupled cavity mode with energy in between the excitons was present. This happened occasionally at low cavity distances, where only few modes are present in the observed spectral region.

**Figure 3:**
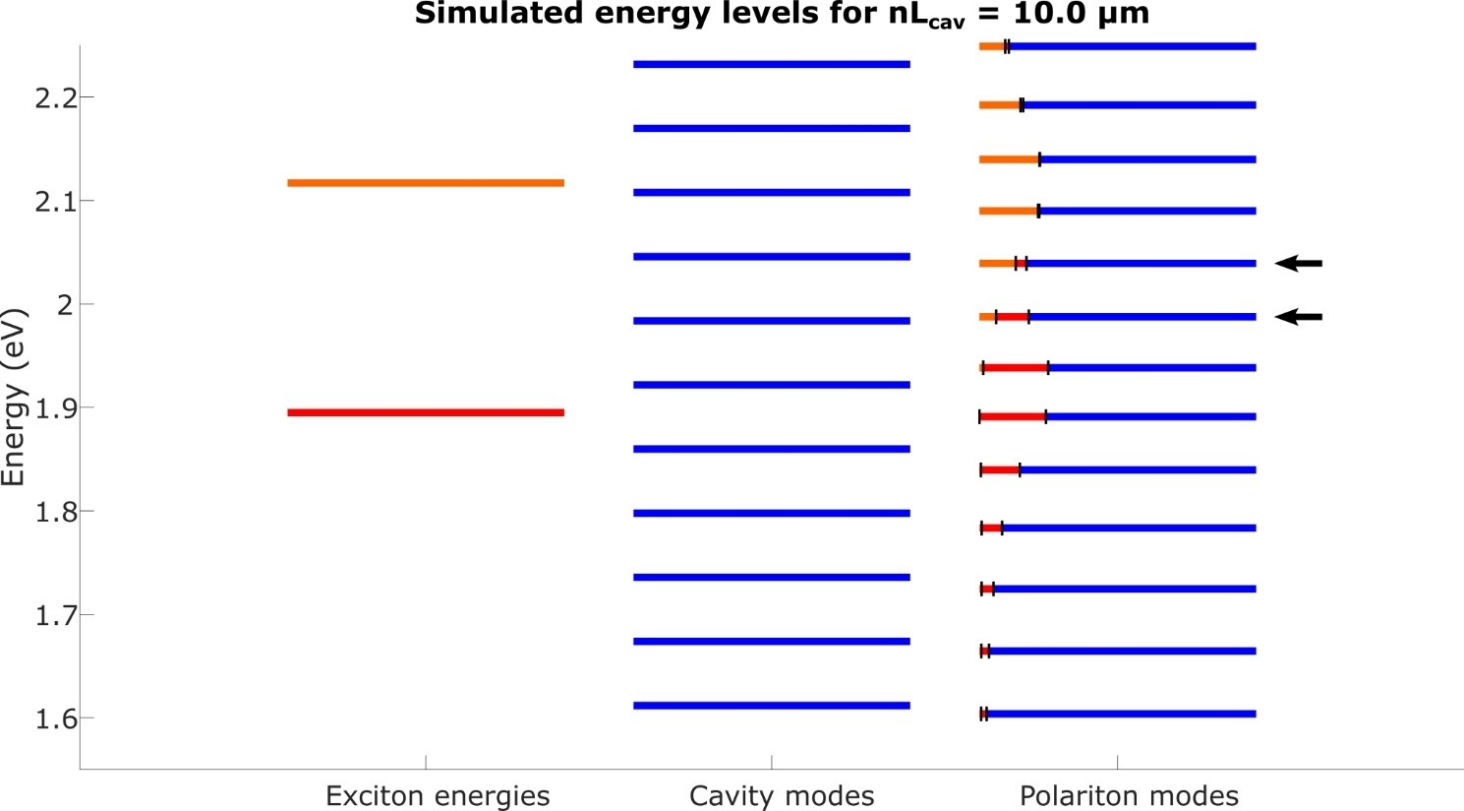
Different energies calculated from $H_{coupled}$ for a cavity with $nL_{cav}=10\;\;\mu m$ : Uncoupled exciton energies (left column; TDBC in orange and BRK in red), uncoupled cavity modes (middle column) and polariton modes for $\hbar g_{TDBC}=35\;\;meV$ and $\hbar g_{BRK}=35\;\;meV$ (right column). For the polariton modes we indicate the respective light and matter fractions by the length of the differently colored sections of the lines (${\left|\alpha^{TDBC}\right|}^{2}$ in orange, ${\left|\alpha^{BRK}\right|}^{2}$ in red and ${\left|\alpha^{light}\right|}^{2}$ in blue). Black arrows mark hybrid polaritons, for which significant contributions from both TDBC and BRK are present.

In Figure 3 we plot the resulting polariton energy levels for an effective cavity length of $nL_{cav}$ =10 μm and for the coupling energies $\hbar g_{TDBC}$ =35 meV and $\hbar g_{BRK}$ =35 meV as a representative example. As a reference, we plot the exciton energies in the left column and the energies of the uncoupled cavity modes in the center column. In the right column, we show the polariton energies of the coupled system. For each polariton, we indicate the fractions of the three different constituents (TDBC, BRK and light), showing that the two polaritons marked with black arrows are indeed hybrid polaritons.

## Conclusions

We have investigated the individual and simultaneous strong optical coupling of the two J‐aggregates TDBC and BRK to a tunable multimode FP resonator with several micrometers mirror separation.

By fitting a phenomenological Hamiltonian to the transmission spectra, we are able to determine the coupling energies as a function of the optical path lengths between the mirrors. We find that $H_{coupled}$ describes our experimental data better than $H_{decoupled}$ . For large cavity distances, the coupling energies decrease as $\hbar g\propto {\left(nL_{cav}\right)}^{-1/2}$ with increasing cavity length in excellent agreement with the expected theoretical behaviour.

The values for the coupling energies are consistent with previous studies, which report coupling energies ranging from 20–160 meV.[^2^, ^3^, ^4^, ^6^, ^51^]

Simultaneous strong coupling paves the road for polariton‐mediated energy transfer, which has important possible applications *e. g*. in solar energy conversion systems and optoelectronic integration. The large mirror separations in our system shows that tunable microcavities could be integrated into microfluidic systems which could be particularly interesting for combining polaritonic chemistry with microchemistry.

## Supporting Information Summary

The authors have cited additional references within the Supporting Information.[^53^, ^54^, ^55^, ^56^, ^57^]

## Conflict of Interests

The authors declare no conflict of interest.

1

## Supporting information

As a service to our authors and readers, this journal provides supporting information supplied by the authors. Such materials are peer reviewed and may be re‐organized for online delivery, but are not copy‐edited or typeset. Technical support issues arising from supporting information (other than missing files) should be addressed to the authors.

Supporting Information

## Data Availability

The data that support the findings of this study are available from the corresponding author upon reasonable request.

## References

[1] D. G. Lidzey , D. Bradley , M. Skolnick , T. Virgili , S. Walker , D. Whittaker , Nature 1998, 395, 53–55.

[2] X. Zhong , T. Chervy , S. Wang , J. George , A. Thomas , J. A. Hutchison , E. Devaux , C. Genet , T. W. Ebbesen , Angew. Chem. 2016, 128, 6310–6314. 10.1002/anie.201600428 27072296

[3] X. Zhong , T. Chervy , L. Zhang , A. Thomas , J. George , C. Genet , J. A. Hutchison , T. W. Ebbesen , Angew. Chem. 2017, 129, 9162–9166. 10.1002/anie.201703539 PMC5575472 28598527

[4] M. Tian , X. Li , Z. Li , X. Zhong , J. Phys. Chem. Lett. 2021, 12, 4944–4950. 34009987 10.1021/acs.jpclett.1c01088

[5] D. M. Coles , N. Somaschi , P. Michetti , C. Clark , P. G. Lagoudakis , P. G. Savvidis , D. G. Lidzey , Nat. Mater. 2014, 13, 712–719. 24793357 10.1038/nmat3950

[6] K. Georgiou , R. Jayaprakash , A. Othonos , D. G. Lidzey , Angew. Chem. 2021, 133, 16797–16803. 10.1002/anie.202105442 PMC8361947 33908681

[7] M. Du , L. A. Martínez-Martínez , R. F. Ribeiro , Z. Hu , V. M. Menon , J. Yuen-Zhou , Chem. Sci. 2018, 9, 6659–6669. 30310599 10.1039/c8sc00171e PMC6115621

[8] T. Pajunpää , F. Nigmatulin , S.-T. Akkanen , H. Fernandez , G. Groenhof , Z. Sun , Phys. Rev. B 2024, 109, 195409.

[9] G. Sandik, J. Feist, F. J. García-Vidal, T. Schwartz, *Nat. Mater*. (published online **2024**). 10.1038/s41563-024-01962-5 39122930

[10] E. Orgiu , J. George , J. Hutchison , E. Devaux , J. Dayen , B. Doudin , F. Stellacci , C. Genet , J. Schachenmayer , C. Genes , Nat. Mater. 2015, 14, 1123–1129. 26366850 10.1038/nmat4392

[11] D. Myers , S. Mukherjee , J. Beaumariage , D. Snoke , M. Steger , L. Pfeiffer , K. West , Phys. Rev. B 2018, 98, 235302.

[12] J. Feist , F. J. Garcia-Vidal , Phys. Rev. Lett. 2015, 114, 196402. 26024185 10.1103/PhysRevLett.114.196402

[13] J. Schachenmayer , C. Genes , E. Tignone , G. Pupillo , Phys. Rev. Lett. 2015, 114, 196403. 26024186 10.1103/PhysRevLett.114.196403

[14] D. Hagenmüller , J. Schachenmayer , S. Schütz , C. Genes , G. Pupillo , Phys. Rev. Lett. 2017, 119, 223601. 29286774 10.1103/PhysRevLett.119.223601

[15] C. Schäfer , M. Ruggenthaler , H. Appel , A. Rubio , Proc. Natl. Acad. Sci. 2019, 116, 4883–4892. 30733295 10.1073/pnas.1814178116 PMC6421448

[16] J. A. Hutchison , T. Schwartz , C. Genet , E. Devaux , T. W. Ebbesen , Angew. Chem. Int. Ed. 2012, 51, 1592–1596. 10.1002/anie.201107033 22234987

[17] A. Thomas , J. George , A. Shalabney , M. Dryzhakov , S. J. Varma , J. Moran , T. Chervy , X. Zhong , E. Devaux , C. Genet , Angew. Chem. 2016, 128, 11634–11638. 10.1002/anie.201605504 PMC5113700 27529831

[18] A. Thomas , L. Lethuillier-Karl , K. Nagarajan , R. M. Vergauwe , J. George , T. Chervy , A. Shalabney , E. Devaux , C. Genet , J. Moran , Science 2019, 363, 615–619. 30733414 10.1126/science.aau7742

[19] W. Ahn , J. F. Triana , F. Recabal , F. Herrera , B. S. Simpkins , Science 2023, 380, 1165–1168. 37319215 10.1126/science.ade7147

[20] B. Munkhbat , M. Wersäll , D. G. Baranov , T. J. Antosiewicz , T. Shegai , Sci. Adv. 2018, 4, eaas9552. 29984306 10.1126/sciadv.aas9552 PMC6035039

[21] J. Lather , P. Bhatt , A. Thomas , T. W. Ebbesen , J. George , Angew. Chem. Int. Ed. 2019, 58, 10635–10638. 10.1002/anie.201905407 PMC6771697 31189028

[22] P. Törmä , W. L. Barnes , Rep. Prog. Phys. 2014, 78, 013901. 25536670 10.1088/0034-4885/78/1/013901

[23] D. Dovzhenko , S. Ryabchuk , Y. P. Rakovich , I. Nabiev , Nanoscale 2018, 10, 3589–3605. 29419830 10.1039/c7nr06917k

[24] J. Flick , N. Rivera , P. Narang , Nanophotonics 2018, 7, 1479–1501.

[25] F. J. Garcia-Vidal , C. Ciuti , T. W. Ebbesen , Science 2021, 373, eabd0336. 34244383 10.1126/science.abd0336

[26] K. Hirai , J. A. Hutchison , H. Uji-i , Chem. Rev. 2023, 123, 8099–8126. 37390295 10.1021/acs.chemrev.2c00748

[27] T. W. Ebbesen , Acc. Chem. Res. 2016, 49, 2403–2412. 27779846 10.1021/acs.accounts.6b00295

[28] C. F. Klingshirn , Semiconductor Optics Third Edition,Springer Berlin Heidelberg New York, 2007, pp. 439–444.

[29] T. Rammler , F. Wackenhut , S. zur Oven-Krockhaus , J. Rapp , K. Forchhammer , K. Harter , A. J. Meixner , J. Biophotonics 2022, 15, e202100136. 34761529 10.1002/jbio.202100136

[30] L. Wang , Q. Liu , F. Wackenhut , M. Brecht , P.-M. Adam , J. Gierschner , A. J. Meixner , J. Chem. Phys. 2022, 156, 014203. 34998354 10.1063/5.0078117

[31] W. M. Takele , F. Wackenhut , Q. Liu , L. Piatkowski , J. Waluk , A. J. Meixner , J. Phys. Chem. C 2021, 125, 14932–14939.

[32] S. Nosrati , F. Wackenhut , C. Kertzscher , M. Brecht , A. J. Meixner , J. Phys. Chem. C 2023, 127, 12152–12159.

[33] D. M. Coles , Y. Yang , Y. Wang , R. T. Grant , R. A. Taylor , S. K. Saikin , A. Aspuru-Guzik , D. G. Lidzey , J. K.-H. Tang , J. M. Smith , Nat. Commun. 2014, 5, 5561. 25429787 10.1038/ncomms6561

[34] D. Urbonas , T. Stöferle , F. Scafirimuto , U. Scherf , R. F. Mahrt , ACS Photonics 2016, 3, 1542–1545.

[35] L. C. Flatten , D. M. Coles , Z. He , D. G. Lidzey , R. A. Taylor , J. H. Warner , J. M. Smith , Nat. Commun. 2017, 8, 14097. 28094281 10.1038/ncomms14097 PMC5247603

[36] S. Betzold , S. Herbst , A. A. Trichet , J. M. Smith , F. Würthner , S. Höfling , C. P. Dietrich , ACS Photonics 2018, 5, 90–94.

[37] R. T. Grant , R. Jayaprakash , D. M. Coles , A. Musser , S. De Liberato , I. D. W. Samuel , G. A. Turnbull , J. Clark , D. G. Lidzey , Opt. Express 2018, 26, 3320–3327. 29401861 10.1364/OE.26.003320

[38] M. Król , K. Lekenta , R. Mirek , K. Łempicka , D. Stephan , K. Nogajewski , M. R. Molas , A. Babiński , M. Potemski , J. Szczytko , Nanoscale 2019, 11, 9574–9579. 31062800 10.1039/c9nr02038a

[39] T. Förster , Ann. Phys., Lpz. 1948, 437, 55–75.

[40] D. L. Dexter , J. Chem. Phys. 1953, 21, 836–850.

[41] F. Würthner , T. E. Kaiser , C. R. Saha-Möller , Angew. Chem. Int. Ed. 2011, 50, 3376–3410. 10.1002/anie.201002307 21442690

[42] J. R. Tischler , M. S. Bradley , Q. Zhang , T. Atay , A. Nurmikko , V. Bulović , Org. Electron. 2007, 8, 94–113.

[43] V. M. Agranovich , M. Litinskaia , D. G. Lidzey , Phys. Rev. B 2003, 67, 085311.

[44] M. Litinskaya , P. Reineker , V. M. Agranovich , J. Lumin. 2004, 110, 364–372.

[45] P. Michetti , G. C. La Rocca , Phys. Rev. B 2008, 77, 195301.

[46] T. Schwartz , J. A. Hutchison , J. Léonard , C. Genet , S. Haacke , T. W. Ebbesen , ChemPhysChem 2013, 14, 125–131. 23233286 10.1002/cphc.201200734

[47] K. Georgiou , R. Jayaprakash , D. Lidzey , J. Phys. Chem. Lett. 2020, 11, 9893–9900. 33170714 10.1021/acs.jpclett.0c02751

[48] M. Fox , Quantum Optics: an introduction, Oxford University Press, Oxford, UK, 2006, pp. 206–209.

[49] A. V. Kavokin , J. J. Baumberg , G. Malpuech , F. P. Laussy , Microcavities Revised Edition, Oxford university press, Oxford, UK, 2007, pp. 177–180.

[50] S. Richter , T. Michalsky , L. Fricke , C. Sturm , H. Franke , M. Grundmann , R. Schmidt-Grund , Appl. Phys. Lett. 2015, 107 (23), 231104.

[51] K. Georgiou , K. E. McGhee , R. Jayaprakash , D. G. Lidzey , J. Chem. Phys. 2021, 154, 124309. 33810682 10.1063/5.0038086

[52] M. Balasubrahmaniyam , C. Genet , T. Schwartz , Phys. Rev. B 2021, 103, L241407.

[53] I. H. Malitson , JOSA 1962, 52, 1377–1379.

[54] M. J. Schnepf , M. Mayer , C. Kuttner , M. Tebbe , D. Wolf , M. Dulle , T. Altantzis , P. Formanek , S. Förster , S. Bals , Nanoscale 2017, 9, 9376–9385. 28656183 10.1039/c7nr02952g

[55] P. Schouwink , H. Berlepsch , L. Dähne , R. Mahrt , Chem. Phys. 2002, 285, 113–120.

[56] S. Wang , T. Chervy , J. George , J. A. Hutchison , C. Genet , T. W. Ebbesen , J. Phys. Chem. Lett. 2014, 5, 1433–1439. 26269990 10.1021/jz5004439

[57] S. Nosrati , T. Rammler , A. J. Meixner , F. Wackenhut , J. Phys. Chem. C 2021, 125, 13024–13032.

