# Supplementary material for: Global Spectral Analysis of Polaritonic Coupling of Multiple Organic Dyes to a Tunable Fabry‐Pérot Resonator Operating with Mirror Separations up to 10 μm
Source: Chemistry. 2025 Mar 5;31(21):e202500344. doi: 10.1002/chem.202500344 (PMC11979686; doi:10.1002/chem.202500344)
Supplement: Supplementary file 1 — Supporting Information [file CHEM-31-e202500344-s001.pdf]

Chemistry–A European Journal

Supporting Information

Global Spectral Analysis of Polaritonic Coupling of Multiple Organic Dyes to a Tunable Fabry-Pérot Resonator Operating with Mirror Separations up to 10 μm

Christoph Kertzscher,* Michael Mauch, Jakob Keck, and Alfred J. Meixner*

Supporting Information

Global Spectral Analysis of Polaritonic Coupling of Multiple Organic Dyes to a Tunable Fabry-Pérot Resonator Operating with Mirror Separations up to 10 µm

Christoph Kertzscher, Michael Mauch, Jakob Keck, Alfred J. Meixner

Table of contents

Author contributions 2

Experimental section 3

Transmission measurement of an empty cavity and determining of the Q-factor 5

Matrix representation for $H_{decoupled}$ and $H_{coupled}$ 7

Fitting procedure 8

Fitting of an individual transmission spectrum 8

Fitting of a series of transmission spectra 8

Cavity distances 8

Coupling energies 10

Differences between the optical path length and distance between the J-aggregate containing layers 12

Influence of the refractive index 12

Calculation of the distance between the J-aggregate containing layers 12

Full false-color maps of the transmission measurements with the fit to $H_{coupled}$ 13

Results for the fits to $H_{decoupled}$ 16

References 19

Author contributions

JK and CK conceived the experiment. MM performed all measurements. MM and CK analyzed the data, including development of the fitting procedure. CK wrote the manuscript with inputs from all authors. AJM supervised the project, contributed to the discussion of the results, reviewed and edited the manuscript.

Experimental section

Preparation of J-aggregate stock solutions: 1 wt% of the J-aggregate TDBC (5,6-dichloro-2-[[5,6-dichloro-1-ethyl-3-(4-sulphobutyl)benzimidazol-2-ylidene]propenyl]-1-ethyl-3-(4-sulphobutyl)benzimidazolium hydroxide) [FEW Chemicals] or BRK (1-(3-Sulfopropyl)-2-(2-{[1-(3-sulfopropyl)naphtho[1,2-d]thiazol-2(1H)-ylidene]methyl}-1-butenyl)naphtha[1,2-d]thiazoliumhydroxide) [ORGANICA Feinchemie] and 3 wt% of Poly(vinyl alcohol) (PVA) [Mowiol 40-88, Sigma-Aldrich] were dissolved in deionized water at room temperature. These solutions were stored in the dark at 8°C for up to two months and spin coated on the respective resonator mirrors as needed.

Spin coating: 20 µl of the J-aggregate stock solution were spin coated at 5000 rpm. For the measurements presented in this work, the TDBC-solution was spin coated onto the top mirror and the BRK-solution was spin coated onto the bottom mirror of the cavity. The resulting films had a thickness between 500 and 600 nm, as measured with a Bruker Dektak XT-A profilometer.

Cavity preparation: The preparation of optical resonators was performed similar to previous works of our group:[1-4] For the bottom mirror, a cleaned glass coverslip was coated with 4 nm Cr, 70 nm Ag and 75 nm Al_2O_3 using electron beam evaporation (EDWARDS EB3 for Cr and Ag; Pfeiffer Vacuum PLS 570 for Al_2O_3). For the top mirror, the same procedure was performed, but with a plano-convex lens (LA1433, Thorlabs; Diameter 25.4 mm, Radius of Curvature 77.3 mm) as a substrate instead of the glass coverslip.

After spin coating the J-aggregate solutions onto the mirrors, the two mirrors were fixed in a custom-build cage system containing three µm-screws and piezo-actuators (KC1-PZ/M, Thorlabs) which were used to adjust the position of the top mirror relative to the bottom mirror. Before the cavity was installed into the custom-built confocal microscope, the µm-screws were used to approach the top mirror towards the bottom mirror until Newton's rings became visible (at the spatial position where the top mirror (lens) is approximately parallel to the bottom mirror (coverslip)), signifying that the mirror separation is on the order of visible wavelengths.

White light transmission spectra at different cavity distances were used to characterize the cavity. For each transmission peak, we determined the Q-factor *via* $Q = \frac{\omega}{\Delta\omega}$ with ω being the angular frequency and $\Delta\omega$ the full width at half maximum (FWHM) of the respective peak, obtained from a fit to a Lorentzian. The resulting (wavelength- and distance-dependent) Q-factors were in the range of 150 to 400 (see Figure S3).

Optical measurements: Optical measurements were performed on a custom-built confocal microscope. For recording transmission spectra, the cavity was illuminated by a KL 1500 LCD lab-lamp (Leica), emitting unpolarized white light. The light was focused onto the back side of the top mirror by a lens (NA approx. 0.1). The transmission light was collected by a 0.5 NA objective, which was focused on the bottom mirror of the cavity and guided to a Princeton Instruments Acton SP-2-500i spectrometer with a thermoelectrically cooled ProEM+ 512B eXcelon camera attached.

This setup can also be used for emission measurements by coupling a laser (532 nm; Coherent COMPASS 215M-75 SL) into the objective and blocking the reflected laser light by two RazorEdge Long Pass (LP) E-Grade 532 filters. For a sketch of the two measurement configurations, see Figure S1.

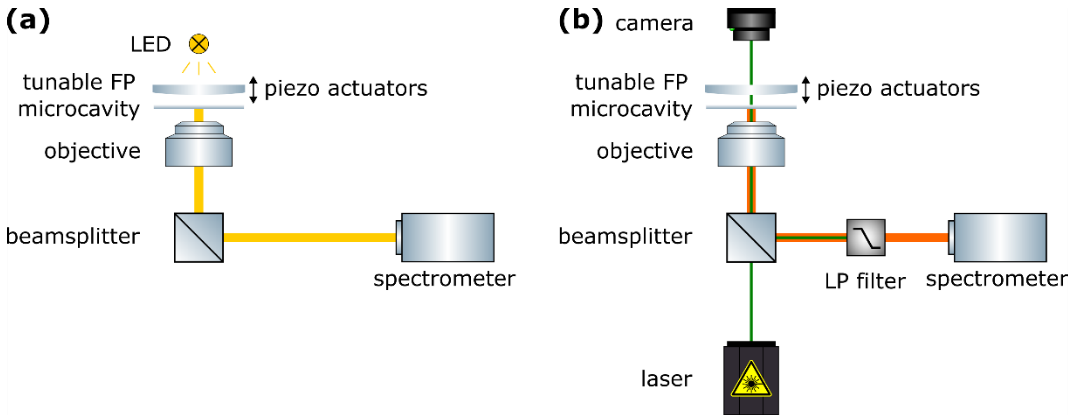

Figure S1. Sketch of the experimental setup configured for transmission measurements shown in (a) and for fluorescence measurements shown in (b).

Free space spectra: Concerning the free space spectra presented in Figure 1: Absorption spectra were measured from PVA thin films using a UV-Vis-NIR spectrophotometer (PerkinElmer Lambda 19). Emission measurements were measured from PVA thin films using the custom-build microscope setup described above.

Software: Data evaluation was performed using MATLAB R2021a.

Transmission measurement of an empty cavity and determining of the Q-factor

In Figure S2 we show the results for a transmission measurement of an empty cavity. The measurement was performed in a way that the two mirrors touched at the start of the measurement. Consequently, for the first seventy spectra, the observed transmission peak changes only slightly. As soon as the mirrors separate, the expected behaviour of linearly red-shifting cavity modes is observed.

The obtained transmission spectra were subsequently used to calculate the Q-factor of the cavity (as described in the experimental section). The obtained values for the Q-factor (for each transmission peak) are displayed in Figure S3. The trend of increasing Q with increasing wavelength for the same mode is explained by the reflectivity of the Ag mirrors, which increases with increasing wavelength for the visible part of the electromagnetic spectrum. The difference between modes is due to the (implicit) relation of the Q-factor to the resonator's lifetime $\tau = \frac{1}{\Delta\omega}$, which is (for constant losses) proportional to the resonator's (round-trip) length. Therefore, the Q-factor increases with increasing cavity distance.

The dips at 555 nm, 655 nm and 760 nm are experimental artifacts due to the "Step and Glue" acquisition mode used to acquire the spectra. The rapid decrease of the Q-factors for wavelengths > 770 nm is probably a consequence of the diminishing detected intensity due to the increasing absorption of Ag at high wavelengths and should be treated as an experimental artifact.

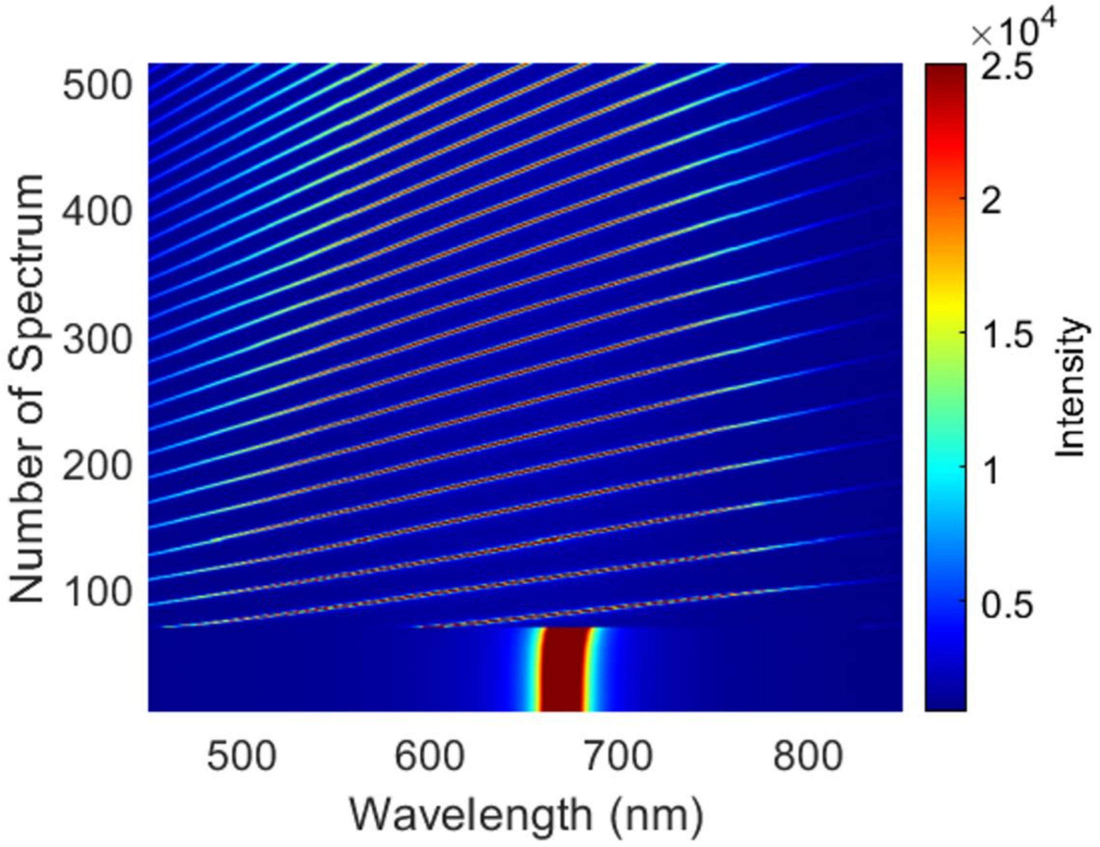

Figure S2. Transmission measurement of an empty cavity. At the start of the measurement, the two mirrors were in contact. We set an upper limit for the color bar to increase contrast.

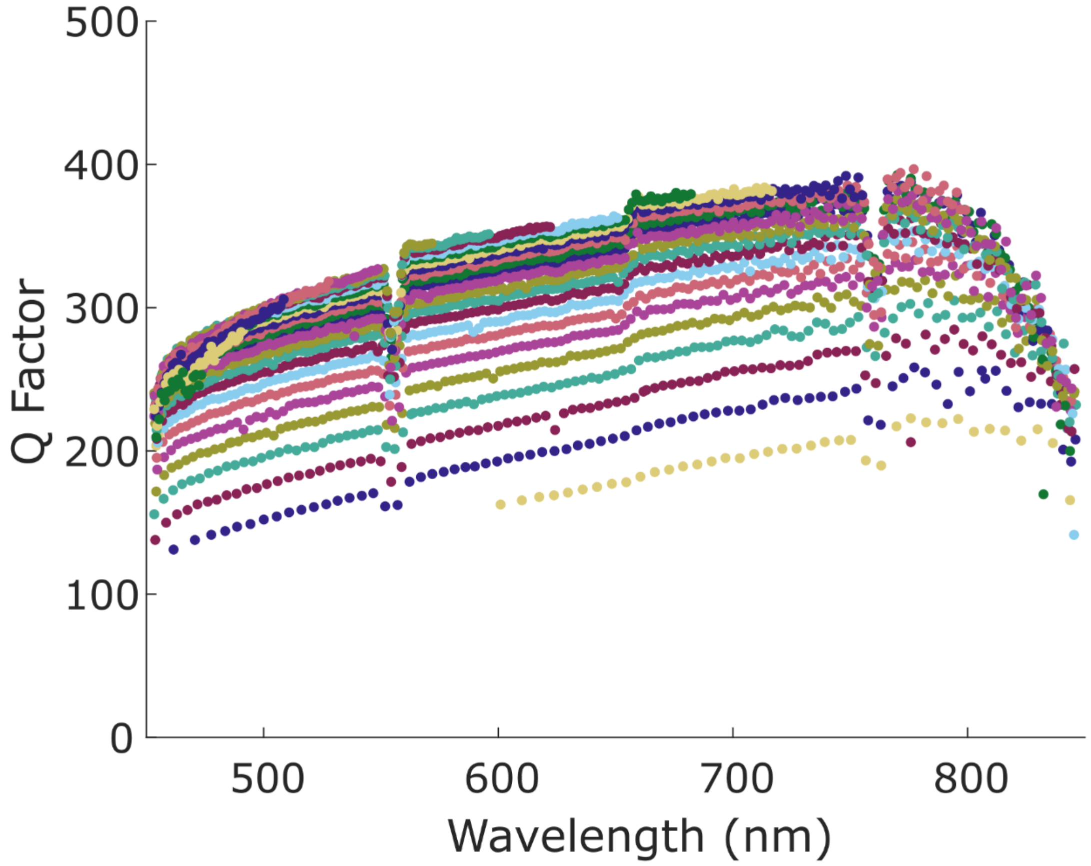

Figure S3. Values for the Q-factor of the cavity, obtained from the transmission peaks shown in Figure S2. Each color represents a specific cavity mode.

Matrix representation for $H_{decoupled}$ and $H_{coupled}$

For $H_{decoupled}$, each cavity mode couples to the excitons independently. For N cavity modes and 2 excitons this results in a matrix of size $3N \times 3N$:

$$H_{decoupled} = \begin{pmatrix} E_{cav,1} & \hbar g_{TDBC} & \hbar g_{BRK} & 0 & 0 & 0 & \cdots & 0 & 0 & 0 \\ \hbar g_{TDBC} & E_{TDBC} & 0 & 0 & 0 & 0 & \cdots & 0 & 0 & 0 \\ \hbar g_{BRK} & 0 & E_{BRK} & 0 & 0 & 0 & \cdots & 0 & 0 & 0 \\ 0 & 0 & 0 & E_{cav,2} & \hbar g_{TDBC} & \hbar g_{BRK} & & & & \\ 0 & 0 & 0 & \hbar g_{TDBC} & E_{TDBC} & 0 & & & & \\ 0 & 0 & 0 & \hbar g_{BRK} & 0 & E_{BRK} & & & & \\ \vdots & \vdots & \vdots & & & & \ddots & & & \\ 0 & 0 & 0 & & & & & E_{cav,N} & \hbar g_{TDBC} & \hbar g_{BRK} \\ 0 & 0 & 0 & & & & & \hbar g_{TDBC} & E_{TDBC} & 0 \\ 0 & 0 & 0 & & & & & \hbar g_{BRK} & 0 & E_{BRK} \end{pmatrix}$$

For comparison, the matrix representation for $H_{coupled}$ is:

$$H_{coupled} = \begin{pmatrix} E_{TDBC} & 0 & \hbar g_{TDBC} & \hbar g_{TDBC} & \cdots & \hbar g_{TDBC} \\ 0 & E_{BRK} & \hbar g_{BRK} & \hbar g_{BRK} & \cdots & \hbar g_{BRK} \\ \hbar g_{TDBC} & \hbar g_{BRK} & E_{cav,1} & 0 & \cdots & 0 \\ \hbar g_{TDBC} & \hbar g_{BRK} & 0 & E_{cav,2} & & 0 \\ \vdots & \vdots & \vdots & & \ddots & 0 \\ \hbar g_{TDBC} & \hbar g_{BRK} & 0 & 0 & 0 & E_{cav,N} \end{pmatrix}$$

Here, the excitons couple with each cavity mode simultaneously.

The main differences between the two Hamiltonians are firstly the different dimensions (for N considered cavity modes and 2 excitons, $size(H_{coupled}) = (N+2) \times (N+2)$ and $size(H_{decoupled}) = 3N \times 3N$) and secondly that for $H_{coupled}$, the resulting polariton modes (as a function of cavity distance) cross the exciton energies, while for $H_{decoupled}$, they do not.

Fitting procedure

To fit the series of transmission spectra to the phenomenological Hamiltonians, we developed a fitting routine in MATLAB R2021a.

Fitting of an individual transmission spectrum

To fit the Hamiltonian to an experimental transmission spectrum, we have constructed a model function for the transmission spectra in which the transmission peaks are described by Lorentzian lineshape functions with peak positions related to the energy eigenvalues of the Hamiltonian. In the Hamiltonian, we fix the exciton energies of the J-aggregates and leave the coupling energies and the cavity length as free parameters. The intensities of the measured transmission peaks depend on the spectrum of the white light source and the spectral properties of the mirrors. To account for this, we have first fitted all peaks in the experimental spectra to Lorentzians and then used the obtained values for the peak intensities and FWHMs for the model function as fixed parameters.

Challenging when performing these fits were local minima in the root-mean-square (RMS) deviation as a function of the fitting parameters: Due to the periodic nature of the cavity modes, the deviation between experimental and modelled transmission spectrum can be periodic with respect to the optical path length. Therefore, the choice of good starting parameters is crucial for obtaining correct fit results.

Fitting of a series of transmission spectra

We subsequently performed the fit for every transmission spectrum of the whole series of spectra and obtained values for the optical path length and the coupling energies for every spectrum. Proceeding from one spectrum to the next, we used the results from the spectrum as starting parameters for fitting the next spectrum in the series.

Cavity distances

Figure S4 shows the cavity distances obtained from the fitting procedures (blue lines) along with the smoothed distances (orange lines) used for the plots in the false-color maps. Smoothing was performed with MATLAB's "smoothdata" function with the "rlowess" method. We want to point out two findings:

- The obtained distances are almost identical for $H_{coupled}$ and $H_{decoupled}$. This agrees very well with the idea that the cavity distances are mainly determined by the numerous peaks which are off-resonant with the exciton peaks, which makes the fit quite robust.
- For the measurement configurations 2M-D, 2M-A and 2M-DA-I, the two mirrors touched at the start of the measurement. This manifested in the obtained cavity distances, which only increase slightly for the first few voltage decreases (for the most pronounced example, see 2M-DA-I).

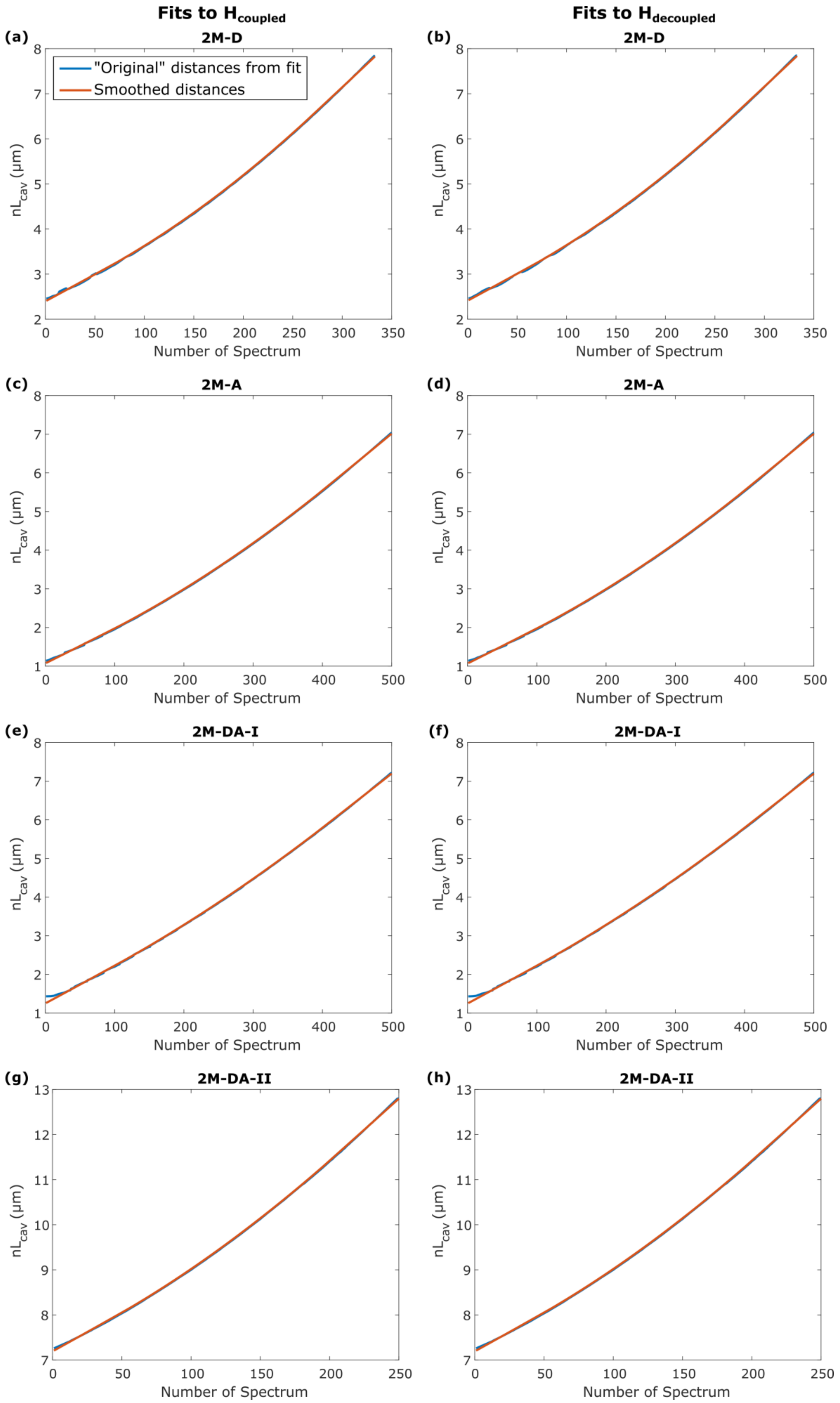

Fits to $H_{coupled}$
Fits to $H_{decoupled}$
(a) 2M-D
(b) 2M-D
(c) 2M-A
(d) 2M-A
(e) 2M-DA-I
(f) 2M-DA-I
(g) 2M-DA-II
(h) 2M-DA-II
"Original" distances from fit
Smoothed distances
nL_{cav} (μm)
Number of Spectrum

Figure S4. Cavity distances obtained from the fitting procedure. Blue lines show the values obtained from the fits; orange lines show the values after smoothing. In the left column, the results for the fit to $H_{coupled}$ are displayed. In the right column, the results for $H_{decoupled}$ are displayed.

Coupling energies

In Figure S5, we show the coupling energies obtained from the fits for all cavity configurations as a function of cavity distance. The orange and red curves represent the expected theoretical evolution of the coupling energy as a function of the cavity length $\hbar g = A \cdot (nL_{cav})^{-1/2}$ for an ideal Fabry-Pérot cavity where we adjusted the parameter A to obtain the best agreement with the obtained coupling energies at high cavity lengths. We obtain larger values of A for the spectral series where only one J-aggregate is present in the cavity (2M-D and 2M-A) as compared to those with both J-aggregates (2M-DA-I/II). Under similar irradiation conditions a possible explanation is that the presence of a second absorbing layer leads to higher absorption losses in the cavity and consequently to a lower number of photons in the cavity modes. Since the coupling energy is proportional to the square root of the photon number in the coupling cavity mode,[5-6] this should lead to a decrease in coupling energy.

In Figure S5 (c) and (d), we observe abrupt steps in the obtained curves at the transition from the measurement 2M-DA-I to 2M-DA-II. Between these two measurements, we used the µm-screws to increase the mirror separation of the cavity by several µm. We cannot be sure that we did not shift the mirrors horizontally to each other with respect to the optical axis. Therefore, these steps might be due to inhomogeneities in the spin coated films, resulting in a slightly lower concentration of TDBC and a slightly higher concentration of BRK for 2M-DA-II as compared to 2M-DA-I for this particular case. Nevertheless, these changes are rather small, and we still obtained good agreement with the theoretical evolution when using the same parameter A for both measurements.

For all cavity configurations displayed in Figure S5, we observe a periodic oscillation of the coupling energies with decreasing amplitude as the cavity length increases. We think that these are due to a subtlety of the setup of the Hamiltonian: We assume that the coupling energy between an exciton and the different cavity modes is the same for all cavity modes (to keep the number of fit parameters reasonable). Hence, we will obtain an average value as the result of the fit as different cavity modes interact with the exciton transition. If now the coupling energies of different cavity modes differ in a systematic way, this can lead to oscillations in the obtained values. We have identified two sources for such differences:

- The spectral width of the excitonic transitions, which was neglected in the Hamiltonian. Here, one would expect different coupling strengths depending on the exact position of the cavity mode in relation to the excitonic maximum. This effect should be averaged out when the free spectral range of the cavity modes is smaller than the width of the excitonic transition.
- The coupling energy also depends on the electric field strength of the cavity mode at the spatial position of the exciton layer. This has been demonstrated experimentally[7-9] and means that exciton-photon coupling is maximal if the exciton layer is located at an antinode of the electric field and completely suppressed if it is located at a node of the electric field. If the cavity length is changed this also changes the spatial structure of the cavity modes and consequently also the coupling efficiency. Since our exciton layers are on the same order of magnitude as the wavelength of the exciton transitions, we expect only a small modulation.

We conclude that for large cavity lengths > approx. 3.5 µm, the simple model we proposed works rather well. For small cavity lengths, a more sophisticated model taking into account the effects mentioned above is needed.

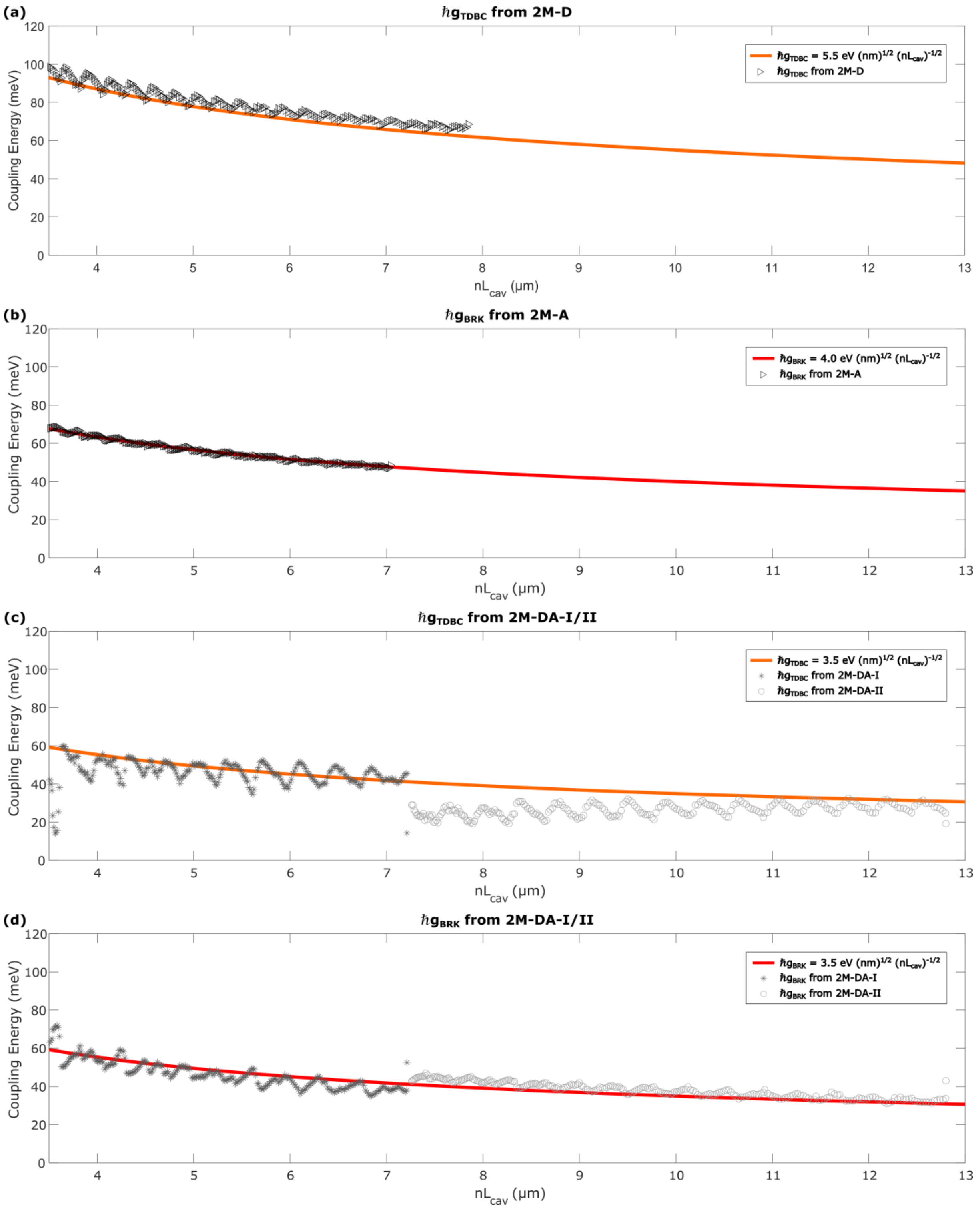

Figure S5: Coupling energies $\hbar g_{TDBC}$ and $\hbar g_{BRK}$ obtained by fitting $H_{coupled}$ to the experimental spectra of 2M-D (a), 2M-A (b) and 2M-DA-I/II (values for $\hbar g_{TDBC}$ in (c) and values for $\hbar g_{BRK}$ in (d)) as a function of the obtained cavity distance. Solid lines show the curves obtained by manually adjusting the function $\hbar g(nL_{cav}) = A \cdot (nL_{cav})^{-1/2}$ for the best agreement with the obtained curves at large distances (TDBC in orange, BRK in red).

Differences between the optical path length and distance between the J-aggregate containing layers

Influence of the refractive index

Concerning the results for the optical path length, we must anticipate a constant offset between optical path length nL_{cav} and "actual" mirror-mirror separation L_{cav} for all measured separations resulting from the refractive index of the Al_2O_3 spacer layer as well as the J-aggregate-containing PVA layers (the space between is filled with air for which $n \approx 1$). If we assume a spacer layer thickness of 75 nm and a PVA layer thickness of around 500 nm for each mirror as well as a refractive index of approximately 1.77 for Al_2O_3[10] and 1.48 for PVA[11] (neglecting the refractive index of the J-aggregates), we expect a difference of around 600 nm between the optical path length nL_{cav} and the "actual" mirror-mirror separation L_{cav} (if the microcavity is filled with both J-aggregates). The effect of the penetration depth of the electromagnetic field into the Ag mirrors is a few tens of nanometers and therefore small compared to the offsets discussed above.

Calculation of the distance between the J-aggregate containing layers

For the measurements involving both J-aggregates (2M-DA-I and 2M-DA-II), we calculated the distance between the J-aggregate containing layers (Table 1). For this purpose, we assumed that these two layers touched at the start of 2M-DA-I. We then subtracted the corresponding value obtained for the optical path length (1.435 µm). This value corresponds rather well to the estimated values for the film thickness of around 500 – 600 nm and the influence of the refractive index on the optical path length of around 600 nm discussed above, which yields an approximate distance of 1.6 – 1.8 µm.

The assumption of touching mirrors at the start of recording the spectral sequence of 2M-DA-I is further confirmed by the experimental data (Figure S4(e)): The obtained optical path lengths for 2M-DA-I show that the cavity distance changed very little for the first few spectra of this measurement.

Full false-color maps of the transmission measurements with the fit to $H_{coupled}$

In Figures S6-S9, we show the entire series of spectra as false-color maps and the results for the fit to $H_{coupled}$ as black circles for the measurement configuration 2M-D (Figure S6), 2M-A (Figure S7), 2M-DA-I (Figure S8) and 2M-DA-II (Figure S9) for cavity lengths > 3.5 µm. Here, we used the coupling energy curves $\hbar g = A \cdot nL_{cav}$ with the values for A displayed in Table 1 of the main text. In the main text (Figure 2), we only show small sections of these false-color maps for better clarity.

A feature in the obtained transmission spectra not discussed in the main text are additional modes of low intensity running parallel to the polariton/cavity modes. These modes are particularly visible at wavelengths greater than 780 nm. We believe that they are caused by the formation of a second cavity between layers that are different from the Ag mirror and therefore have significantly lower reflectivity.

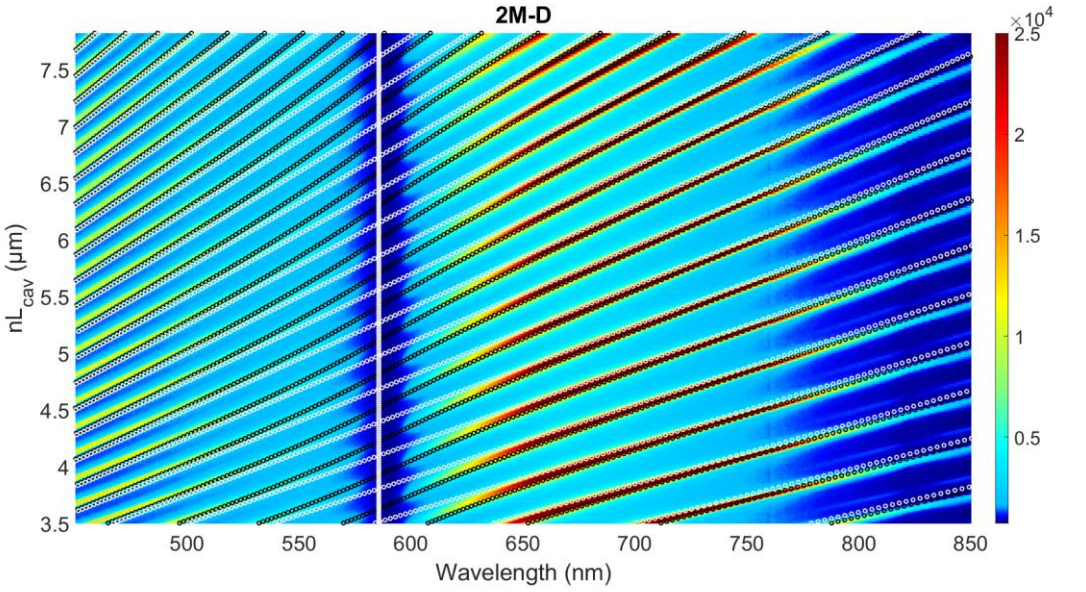

Figure S6. Transmission spectra plotted as false-color maps for 2M-D. Black circles show the fit to $H_{coupled}$. For comparison, white circles show the corresponding uncoupled excitons and cavity modes. The spectra are plotted against the cavity distances from the fit.

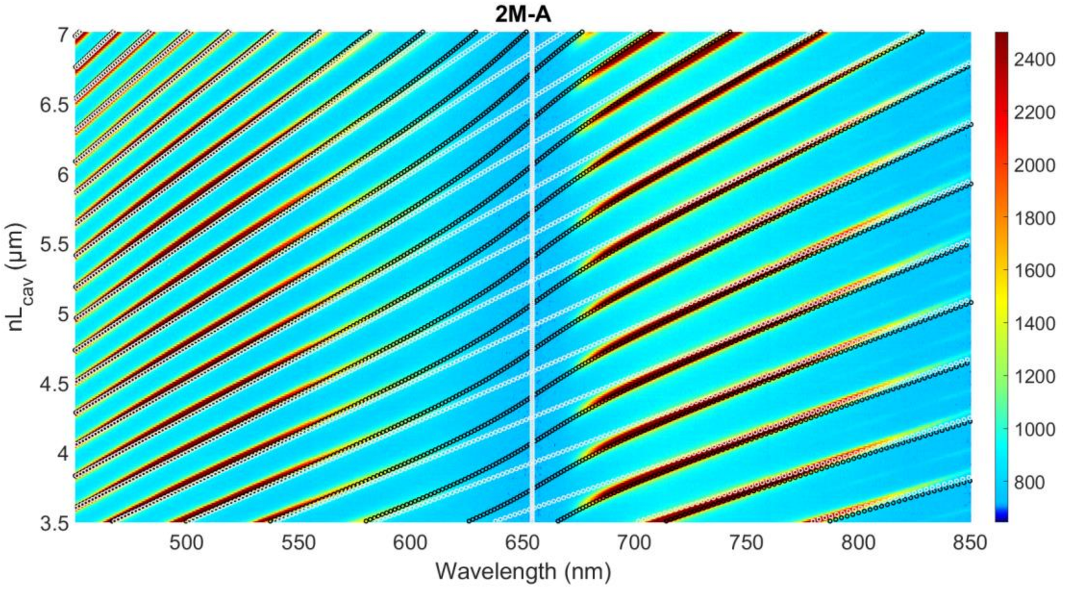

Figure S7. Transmission spectra plotted as false-color maps for 2M-A. Black circles show the fit to $H_{coupled}$. For comparison, white circles show the corresponding uncoupled excitons and cavity modes. The spectra are plotted against the cavity distances from the fit.

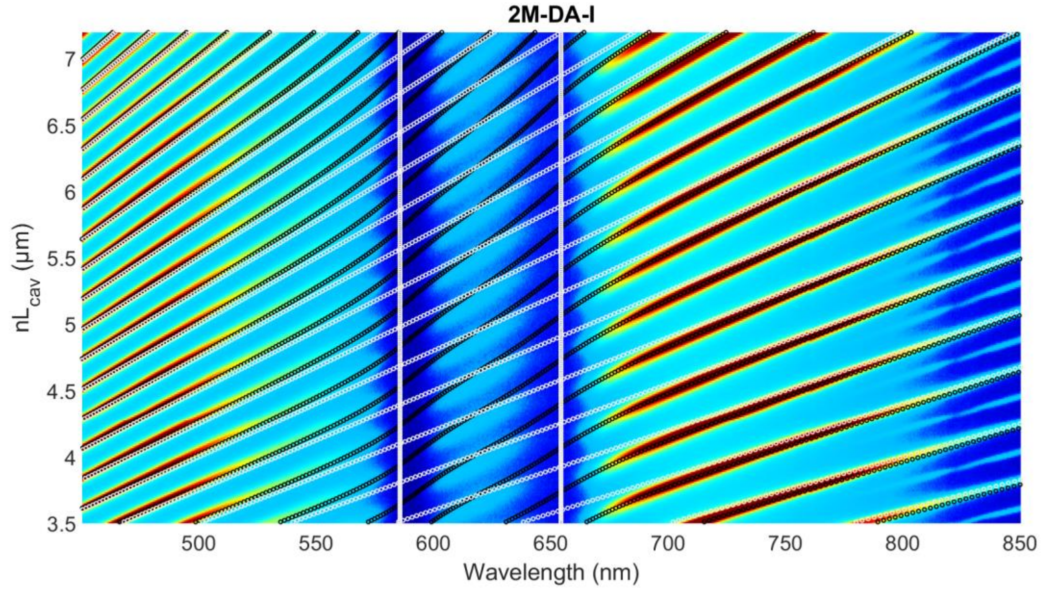

Figure S8. Transmission spectra plotted as false-color maps for 2M-DA-I. Black circles show the fit to $H_{coupled}$. For comparison, white circles show the corresponding uncoupled excitons and cavity modes. The spectra are plotted against the cavity distances from the fit.

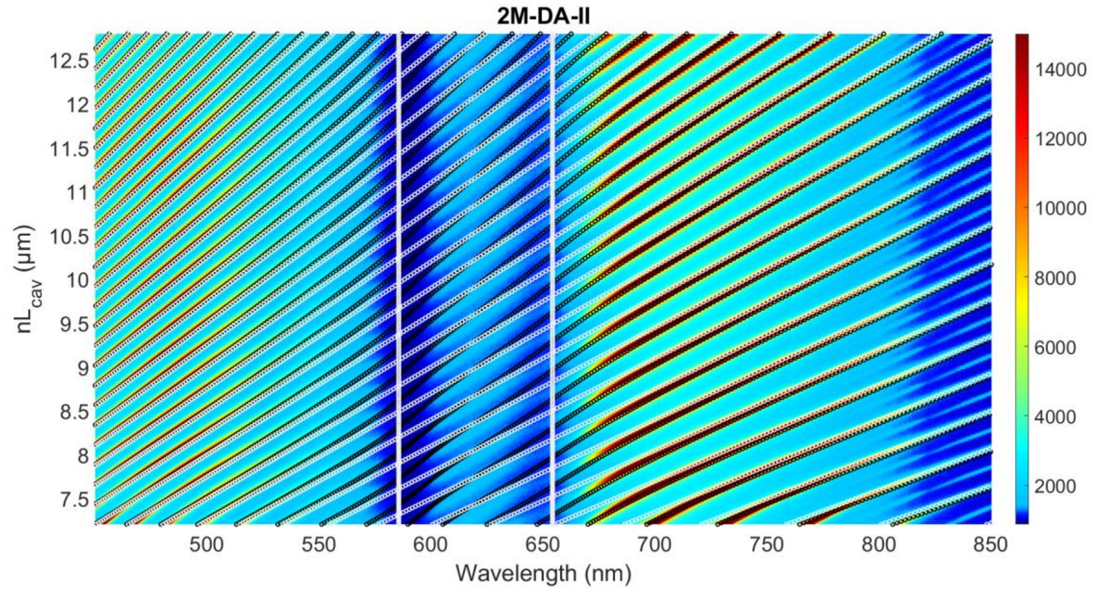

Figure S9. Transmission spectra plotted as false-color maps for 2M-DA-II. Black circles show the fit to $H_{coupled}$. For comparison, white circles show the corresponding uncoupled excitons and cavity modes. The spectra are plotted against the cavity distances from the fit.

Results for the fits to $H_{decoupled}$

Figures S10-S13 show the same false-color maps as in Figures S6-S9, but with $H_{decoupled}$ used for the fits instead of $H_{coupled}$. For the coupling energies, we obtained slightly different values and chose

- A = 5.0 eV (nm)$^{-1/2}$ (TDBC) for 2M-D,
- A = 4.0 eV (nm)$^{-1/2}$ (BRK) for 2M-A,
- A = 4.0 eV (nm)$^{-1/2}$ (TDBC) and A = 3.0 eV (nm)$^{-1/2}$ (BRK) for 2M-DA-I and 2M-DA-II.

The agreement between experimental data and the best fit to the Hamiltonian is significantly worse here. This is most apparent for the spectral region around the TDBC exciton lines, where the resulting polariton modes change far more steeply than the experimental transmission maxima when approaching the exciton line.

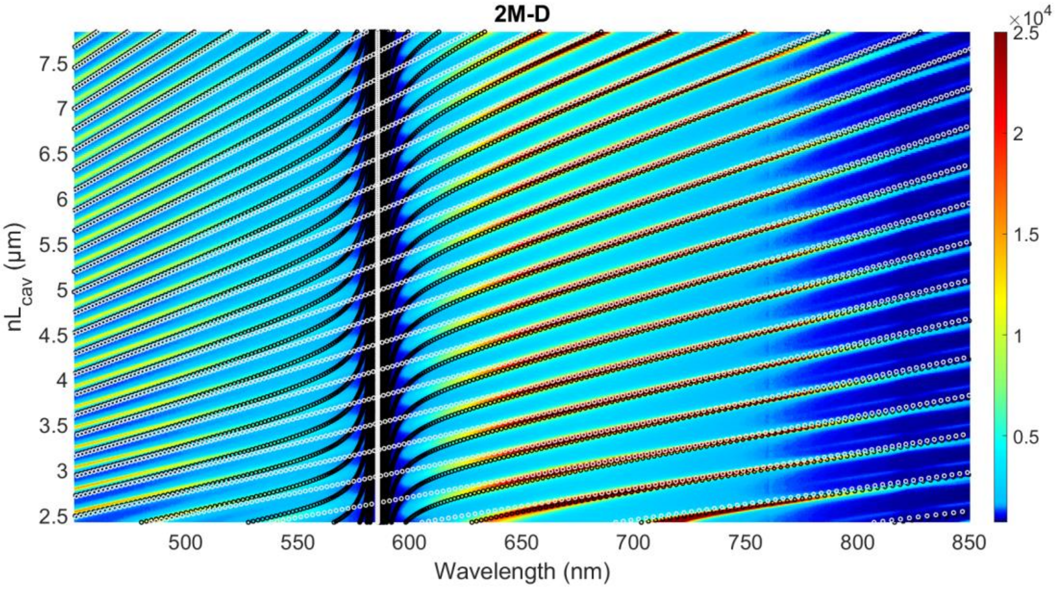

Figure S10. Transmission spectra plotted as false-color maps for 2M-D. Black circles show the fit to $H_{decoupled}$. For comparison, white circles show the corresponding uncoupled excitons and cavity modes. The spectra are plotted against the cavity distances from the fit.

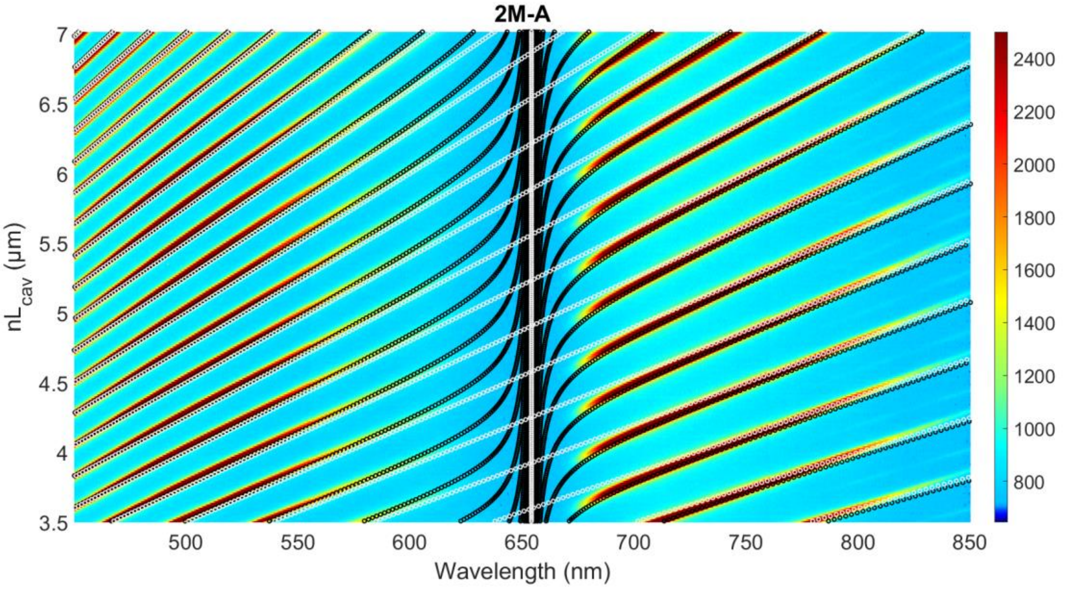

Figure S11. Transmission spectra plotted as false-color maps for 2M-A. Black circles show the fit to $H_{decoupled}$. For comparison, white circles show the corresponding uncoupled excitons and cavity modes. The spectra are plotted against the cavity distances from the fit.

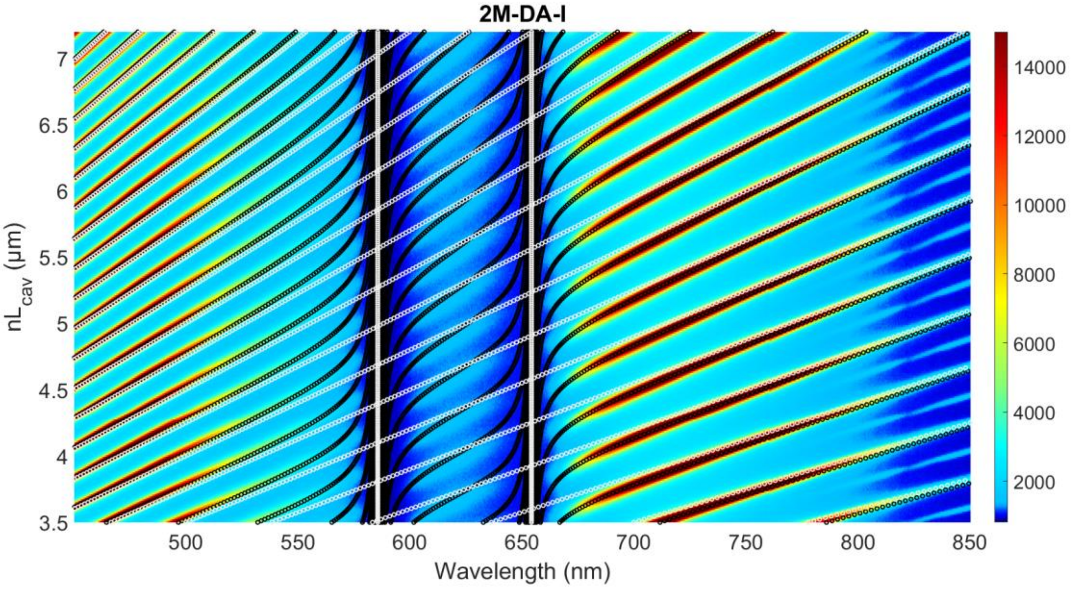

Figure S12. Transmission spectra plotted as false-color maps for 2M-DA-I. Black circles show the fit to $H_{decoupled}$. For comparison, white circles show the corresponding uncoupled excitons and cavity modes. The spectra are plotted against the cavity distances from the fit.

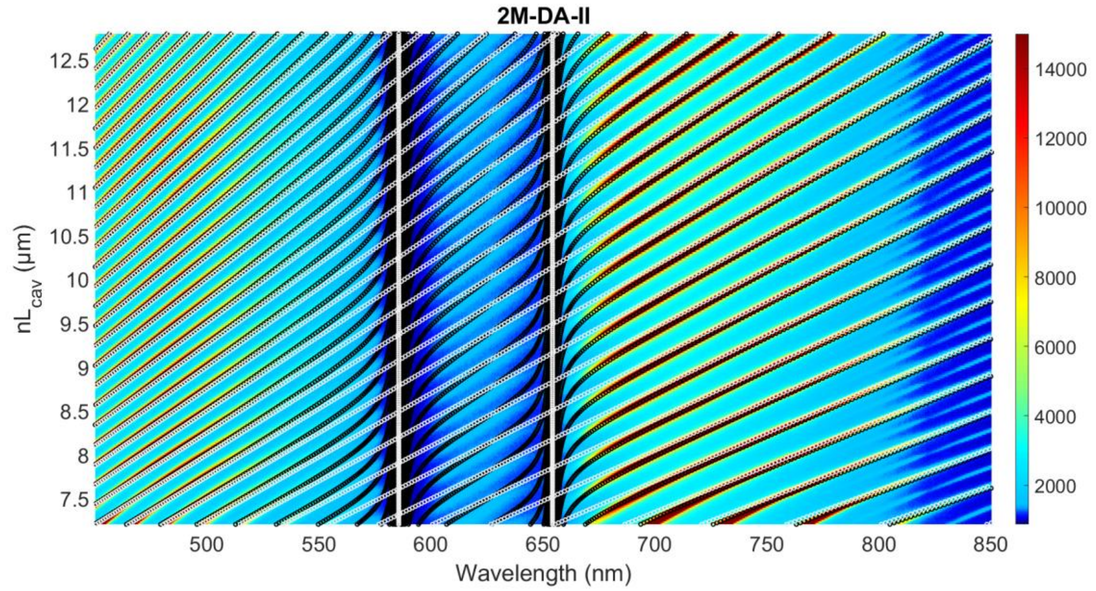

Figure S13. Transmission spectra plotted as false-color maps for 2M-DA-II. Black circles show the fit to $H_{decoupled}$. For comparison, white circles show the corresponding uncoupled excitons and cavity modes. The spectra are plotted against the cavity distances from the fit.

References

[1] T. Rammler, F. Wackenhut, S. zur Oven-Krockhaus, J. Rapp, K. Forchhammer, K. Harter, A. J. Meixner, *Journal of Biophotonics* **2022**, *15*, e202100136.

[2] L. Wang, Q. Liu, F. Wackenhut, M. Brecht, P.-M. Adam, J. Gierschner, A. J. Meixner, *The journal of chemical physics* **2022**, *156*.

[3] W. M. Takele, F. Wackenhut, Q. Liu, L. Piatkowski, J. Waluk, A. J. Meixner, *The Journal of Physical Chemistry C* **2021**, *125*, 14932-14939.

[4] S. Nosrati, F. Wackenhut, C. Kertzscher, M. Brecht, A. J. Meixner, *The Journal of Physical Chemistry C* **2023**, *127*, 12152-12159.

[5] A. M. Fox, *Quantum optics: an introduction, Vol. 15*, Oxford University Press, USA, **2006**.

[6] A. V. Kavokin, J. J. Baumberg, G. Malpuech, F. P. Laussy, *Microcavities, Vol. 21*, Oxford university press, **2017**.

[7] P. Schouwink, H. Berlepsch, L. Dähne, R. Mahrt, *Chemical physics* **2002**, *285*, 113-120.

[8] S. Wang, T. Chervy, J. George, J. A. Hutchison, C. Genet, T. W. Ebbesen, *The journal of physical chemistry letters* **2014**, *5*, 1433-1439.

[9] S. Nosrati, T. Rammler, A. J. Meixner, F. Wackenhut, *The Journal of Physical Chemistry C* **2021**, *125*, 13024-13032.

[10] I. H. Malitson, *JOSA* **1962**, *52*, 1377-1379.

[11] M. J. Schnepf, M. Mayer, C. Kuttner, M. Tebbe, D. Wolf, M. Dulle, T. Altantzis, P. Formanek, S. Förster, S. Bals, *Nanoscale* **2017**, *9*, 9376-9385.
